# Alcohol consumption and epigenetic age acceleration in young adults

**DOI:** 10.18632/aging.204467

**Published:** 2023-01-05

**Authors:** Drew R. Nannini, Brian T. Joyce, Yinan Zheng, Tao Gao, Jun Wang, Lei Liu, David R. Jacobs, Pamela J. Schreiner, Chunyu Liu, Qi Dai, Steve Horvath, Ake T. Lu, Kristine Yaffe, Philip Greenland, Donald M. Lloyd-Jones, Lifang Hou

**Affiliations:** 1 Department of Preventive Medicine, Northwestern University Feinberg School of Medicine, Chicago, IL 60611, USA; 2 Division of Biostatistics, Washington University, St. Louis, MO 63110, USA; 3 Division of Epidemiology and Community Health, School of Public Health, University of Minnesota, Minneapolis, MN 55455, USA; 4 Department of Biostatistics, Boston University School of Public Health, Boston, MA 02118, USA; 5 Department of Medicine, Division of Epidemiology, Vanderbilt Epidemiology Center, Vanderbilt University School of Medicine, Vanderbilt-Ingram Cancer Center, Vanderbilt University Medical Center, Nashville, TN 37232, USA; 6 Department of Human Genetics, David Geffen School of Medicine, University of California, Los Angeles, CA 90095, USA; 7 Department of Biostatistics, Fielding School of Public Health, University of California, Los Angeles, CA 90095, USA; 8 University of California at San Francisco School of Medicine, San Francisco, CA 94143, USA; 9 Department of Medicine, Northwestern University Feinberg School of Medicine, Chicago, IL 60611, USA

**Keywords:** alcohol, epigenetic age, DNA methylation, lifetime alcohol consumption, binge drinking

## Abstract

Alcohol is a widely consumed substance in the United States, however its effect on aging remains understudied. In this study of young adults, we examined whether cumulative alcohol consumption, i.e., alcohol years of beer, liquor, wine, and total alcohol, and recent binge drinking, were associated with four measures of age-related epigenetic changes via blood DNA methylation. A random subset of study participants in the Coronary Artery Risk Development in Young Adults Study underwent DNA methylation profiling using the Illumina MethylationEPIC Beadchip. Participants with alcohol consumption and methylation data at examination years 15 (*n* = 1,030) and 20 (*n* = 945) were included. Liquor and total alcohol consumption were associated with a 0.31-year (*P* = 0.002) and a 0.12-year (*P* = 0.013) greater GrimAge acceleration (GAA) per additional five alcohol years, while beer and wine consumption observed marginal (*P* = 0.075) and no associations (*P* = 0.359) with GAA, respectively. Any recent binge drinking and the number of days of binge drinking were associated with a 1.38-year (*P* < 0.001) and a 0.15-year (*P* < 0.001) higher GAA, respectively. We observed statistical interactions between cumulative beer (*P* < 0.001) and total alcohol (*P* = 0.004) consumption with chronological age, with younger participants exhibiting a higher average in GAA compared to older participants. No associations were observed with the other measures of epigenetic aging. These results suggest cumulative liquor and total alcohol consumption and recent binge drinking may alter age-related epigenetic changes as captured by GAA. With the increasing aging population and widespread consumption of alcohol, these findings may have potential implications for lifestyle modification to promote healthy aging.

## INTRODUCTION

Alcohol is widely consumed in the United States, with 86% and 27% of individuals 18 years of age or older having ever consumed alcohol and having binged alcohol in the past month, respectively [1]. The effects of alcohol on health are complex, with moderate consumption associated with lower risks of diabetes and ischemic heart disease, although the risk of these diseases increase with greater consumption [2]. In comparison, the risk of cancer, including oral, liver, female breast, and colorectal, monotonically increases with greater alcohol consumption [2, 3]. In addition to the amount of alcohol consumed, the type of alcohol has demonstrated different effects on health outcomes. For example, a meta-analysis observed increased risk of lung cancer with greater consumption of beer and liquor, whereas modest consumption of wine was associated with lower risk [4]. Additionally, alcohol consumption has been shown to differ by demographic characteristics, with declining consumption with increasing age and higher consumption among males and self-reported White individuals [5, 6]. While previous studies identified associations between alcohol and age-related health outcomes, studies investigating the effect, type, and pattern of alcohol consumption, in conjunction with demographic characteristics, on the aging process at a molecular level remain limited. Furthermore, a better understanding of the molecular and biological processes associated with alcohol consumption, both chronically and acutely, may improve our understanding of its complex relationship with health and potentially provide new modalities to screen for and prevent age- and alcohol-related chronic diseases.

Molecular markers of biological aging have provided insights into the aging process and age-related conditions. As an indicator of biological age estimated from DNA methylation levels, epigenetic age captures age-related changes to the epigenome and is highly correlated with chronological age [7]. Furthermore, the divergence between epigenetic age and chronological age is used to estimate epigenetic age acceleration (EAA), with positive values indicating an older epigenetic age relative to chronological age and vice versa. Numerous epigenetic metrics of biological age have been developed, including those by Horvath (intrinsic epigenetic age), Hannum (extrinsic epigenetic age), Levine (PhenoAge), and Lu (GrimAge), and have been associated with lifestyle factors, age-related diseases, physical functioning, and lifespan [7–10].

Studies have previously identified associations between alcohol consumption and several EAA metrics [11–16]. In addition, alcohol use disorders have been associated with epigenetic aging in blood and liver tissues, suggesting potential alcohol induced tissue-specific age related changes [17, 18]. Despite these findings, few studies have examined both cumulative and binged alcohol consumption, and the type of alcohol consumed, on epigenetic age-related changes. Therefore, we investigated the associations between cumulative and binged alcohol consumption, as well as alcohol type, on four measures of EAA in the Coronary Artery Risk Development in Young Adults (CARDIA) Study.

## RESULTS

### Sample characteristics

Characteristics of study participants who underwent DNA methylation profiling at examination year (Y) 15 and Y20 were previously found to be largely similar to those participants who did not undergo DNA methylation profiling [19]. Table 1 presents descriptive characteristics for the 1,030 and 945 participants who underwent DNA methylation profiling by binge drinking status at Y15 and Y20, respectively. Among participants who drink, those who recently binge drank had an average of 24.6 ± 22.6 and 31.3 ± 27.5 total alcohol years at Y15 and Y20, compared to an average of 7.1 ± 8.4 and 10.8 ± 17.0 total alcohol years among those who did not recently binge drink, respectively. At both examination years, participants who recently binge drank exhibited a higher GAA compared to non-drinkers and participants who did not recently binge drink (*P* < 0.001), while the other EAA metrics did not show a similar trend.

**Table 1:** Descriptive statistics of study participants at examination years 15 and 20.

***N***	**Year 15**				**Year 20**			
	**Non-drinkers**	**No recent binge**	**Recent binge**	***P***	**Non-drinkers**	**No recent binge**	**Recent binge**	***P***
	**178**	**592**	**260**		**155**	**566**	**224**	
Female, *n*	112 (62.9%)	336 (56.8%)	78 (30.0%)	<0.001	95 (61.3%)	308 (45.6%)	80 (35.7%)	<0.001
Race, *n*				0.003				0.006
Black	91 (51.1%)	235 (39.7%)	91 (35.0%)		81 (52.3%)	219 (38.7%)	85 (38.0%)	
White	87 (48.9%)	357 60.3%)	169 (65.0%)		74 (47.7%)	347 (61.3%)	139 (62.0%)	
Chronological Age, years	40.1 (3.8)	40.7 (3.5)	40.0 (3.3)	0.019	45.2 (3.6)	45.6 (3.6)	45.0 (3.3)	0.067
IEAA, years	0.1 (4.2)	0.1 (4.2)	–0.2 (4.6)	0.656	0.6 (4.5)	–0.1 (4.6)	–0.2 (3.9)	0.220
EEAA, years	–0.1 (5.2)	–0.1 (5.2)	0.1 (5.2)	0.893	0.7 (5.2)	–0.1 (5.1)	–0.1 (4.8)	0.160
PAA, years	–0.5 (5.8)	0.0 (6.0)	0.3 (6.3)	0.437	0.4 (6.0)	–0.2 (6.0)	0.3 (6.2)	0.406
GAA, years	–1.3 (3.8)	–0.3 (4.4)	1.4 (5.0)	<0.001	–1.4 (4.0)	–0.3 (4.4)	1.6 (4.8)	<0.001
Education, years	15.0 (2.3)	15.2 (2.6)	14.9 (2.6)	0.304	14.8 (2.4)	15.2 (2.5)	14.8 (2.5)	0.091
Center, *n*				<0.001				0.001
Birmingham, AL	68 (38.2%)	134 (22.6%)	50 (19.2%)		56 (36.1%)	118 (20.8%)	44 (19.6%)	
Chicago, IL	30 (16.9%)	127 (21.5%)	66 (25.4%)		28 (18.1%)	118 (20.8%)	60 (26.8%)	
Minneapolis, MN	38 (21.3%)	157 (26.5%)	81 (31.2%)		37 (23.9%)	163 (28.9%)	56 (25.0%)	
Oakland, CA	42 (23.6%)	174 (29.4%)	63 (24.2%)		34 (21.9%)	167 (29.5%)	64 (28.6%)	
Pack years of smoking, year	1.5 (4.7)	4.0 (7.7)	6.6 (9.9)	<0.001	1.7 (5.0)	4.8 (9.1)	6.6 (9.3)	<0.001
Physical activity, intensity score	278.1 (246.9)	328.3 (253.3)	449.0 (311.1)	<0.001	288.7 (264.6)	337.4 (267.6)	423.8 (289.1)	<0.001
BMI, kg/m^2^	29.6 (6.8)	28.4 (6.6)	28.1 (4.9)	0.031	30.7 (7.1)	29.2 (6.7)	28.6 (5.2)	0.008
Alcohol years, years								
Beer	0 (0)	3.6 (6.1)	15.5 (17.4)	<0.001	0 (0)	5.6 (13.2)	17.6 (18.9)	<0.001
Liquor	0 (0)	1.4 (2.9)	5.2 (8.9)	<0.001	0 (0)	2.2 (5.5)	6.9 (11.0)	<0.001
Wine	0 (0)	2.2 (3.8)	3.9 (6.6)	<0.001	0 (0)	3.0 (5.0)	6.8 (11.3)	<0.001
Total Alcohol	0 (0)	7.1 (8.4)	24.6 (22.6)	<0.001	0 (0)	10.8 (17.0)	31.3 (27.5)	<0.001
Binge in last 30 days, days	0 (0)	0 (0)	4.1 (5.5)	<0.001	0 (0)	0 (0)	3.7 (4.8)	<0.001

All statistics shown are mean and standard deviation, except for sex, race, and center, which are shown as number of participants and percentages. Abbreviations: IEAA: intrinsic epigenetic age acceleration; EEAA: extrinsic epigenetic age acceleration; PAA: PhenoAge acceleration; GAA: GrimAge acceleration; BMI: body mass index.

### Cumulative alcohol consumption on epigenetic age acceleration

Table 2 presents results for the associations between the cumulative alcohol consumption variables and EAA. After adjusting for covariates, beer years (*P* = 0.035 and *P* = 0.001) and liquor years (*P* = 0.003 and *P* < 0.001) were positively associated with GAA at Y15 and Y20, respectively. Each additional five beer years was associated with a 0.12-year [95% CI: 0.01, 0.22] and a 0.15-year [95% CI: 0.06, 0.24] higher GAA at Y15 and Y20, respectively. Each additional five liquor years was associated with a 0.32-year [95% CI: 0.11, 0.54] and a 0.33-year [95% CI: 0.16, 0.50] higher GAA at Y15 and Y20, respectively. Total alcohol years were positively associated with GAA (*P* = 0.005 and *P* < 0.001), with a 0.11-year [95% CI: 0.03, 0.19] and 0.13-year [95% CI: 0.07, 0.19] higher GAA per five alcohol years at Y15 and Y20, respectively. Wine years were not associated with GAA at either Y15 or Y20. Findings from GEE analyses yielded similar results as Y15 and Y20. IEAA (intrinsic epigenetic age acceleration), EEAA (extrinsic epigenetic age acceleration), and PAA (PhenoAge acceleration) were not associated with the cumulative alcohol consumption variables. Restricting to study participants with complete follow up yielded consistent conclusions (Supplementary Table 1). We observed similar findings when alcohol consumption was categorized into non-drinkers, low, intermediate, and high alcohol consumption (Supplementary Tables 2–4). Similar results as total alcohol years were observed using the cumulative amount of absolute alcohol consumed (Supplementary Table 5). When analyzing weekly alcohol consumption, only liquor consumption was positively associated with GAA (Supplementary Table 6). We observed correlations between beer, liquor, and total alcohol years and several GrimAge surrogate biomarkers of blood plasma proteins at Y15 and Y20, including adrenomedullin, leptin, and plasminogen activation inhibitor 1 (PAI-1) (Supplementary Figures 1, 2). Telomere length estimates were derived from DNA methylation and no cumulative alcohol variable was associated with telomere length (Supplementary Table 7).

**Table 2:** Analysis results for the association between cumulative alcohol consumption and EAA at examination years 15 and 20.

	**Year 15**		**Year 20**		**GEE**	
	**β [95% CI]**	***P***	**β [95% CI]**	***P***	**β [95% CI]**	***P***
Beer Years						
IEAA	–0.04 [–0.17, 0.09]	0.526	–0.08 [–0.19, 0.02]	0.129	–0.07 [–0.17, 0.04]	0.216
EEAA	0.01 [–0.15, 0.16]	0.909	–0.03 [–0.15, 0.09]	0.600	–0.02 [–0.13, 0.09]	0.758
PAA	0.11 [–0.07, 0.29]	0.241	0.05 [–0.10, 0.19]	0.516	0.07 [–0.05, 0.19]	0.275
GAA	0.12 [0.01, 0.22]	0.035	0.15 [0.06, 0.24]	0.001	0.13 [–0.01, 0.27]	0.075
Liquor Years						
IEAA	0.12 [–0.13, 0.38]	0.344	–0.11 [–0.32, 0.10]	0.306	–0.02 [–0.22, 0.18]	0.832
EEAA	–0.13 [–0.44, 0.17]	0.396	–0.08 [–0.30, 0.15]	0.512	–0.11 [–0.29, 0.07]	0.247
PAA	0.19 [–0.16, 0.55]	0.291	0.13 [–0.15, 0.41]	0.372	0.14 [–0.13, 0.41]	0.313
GAA	0.32 [0.11, 0.54]	0.003	0.33 [0.16, 0.50]	<0.001	0.31 [0.11, 0.51]	0.002
Wine Years						
IEAA	0.17 [–0.13, 0.48]	0.265	0.03 [–0.19, 0.24]	0.813	0.08 [–0.08, 0.23]	0.328
EEAA	–0.01 [–0.38, 0.35]	0.952	–0.06 [–0.29, 0.17]	0.610	–0.05 [–0.26, 0.16]	0.645
PAA	0.18 [–0.24, 0.61]	0.405	0.02 [–0.27, 0.31]	0.892	0.07 [–0.22, 0.35]	0.649
GAA	0.08 [–0.18, 0.33]	0.546	0.11 [–0.07, 0.28]	0.228	0.09 [–0.11, 0.30]	0.359
Total Alcohol Years						
IEAA	0.01 [–0.08, 0.10]	0.820	–0.05 [–0.12, 0.02]	0.182	–0.03 [–0.10, 0.04]	0.436
EEAA	–0.01 [–0.13, 0.10]	0.806	–0.03 [–0.11, 0.05]	0.438	–0.03 [–0.10, 0.05]	0.455
PAA	0.10 [–0.03, 0.23]	0.138	0.04 [–0.06, 0.14]	0.414	0.06 [–0.03, 0.15]	0.202
GAA	0.11 [0.03, 0.19]	0.005	0.13 [0.07, 0.19]	<0.001	0.12 [0.02, 0.21]	0.013

Results are adjusted for chronological age, sex, race, center, education, pack years of smoking, BMI, and physical activity. Beta coefficients represent the gain in EAA for each additional 5 alcohol years. Abbreviations: IEAA: intrinsic epigenetic age acceleration; EEAA: extrinsic epigenetic age acceleration; PAA: PhenoAge acceleration; GAA: GrimAge acceleration.

**Figure 1:**
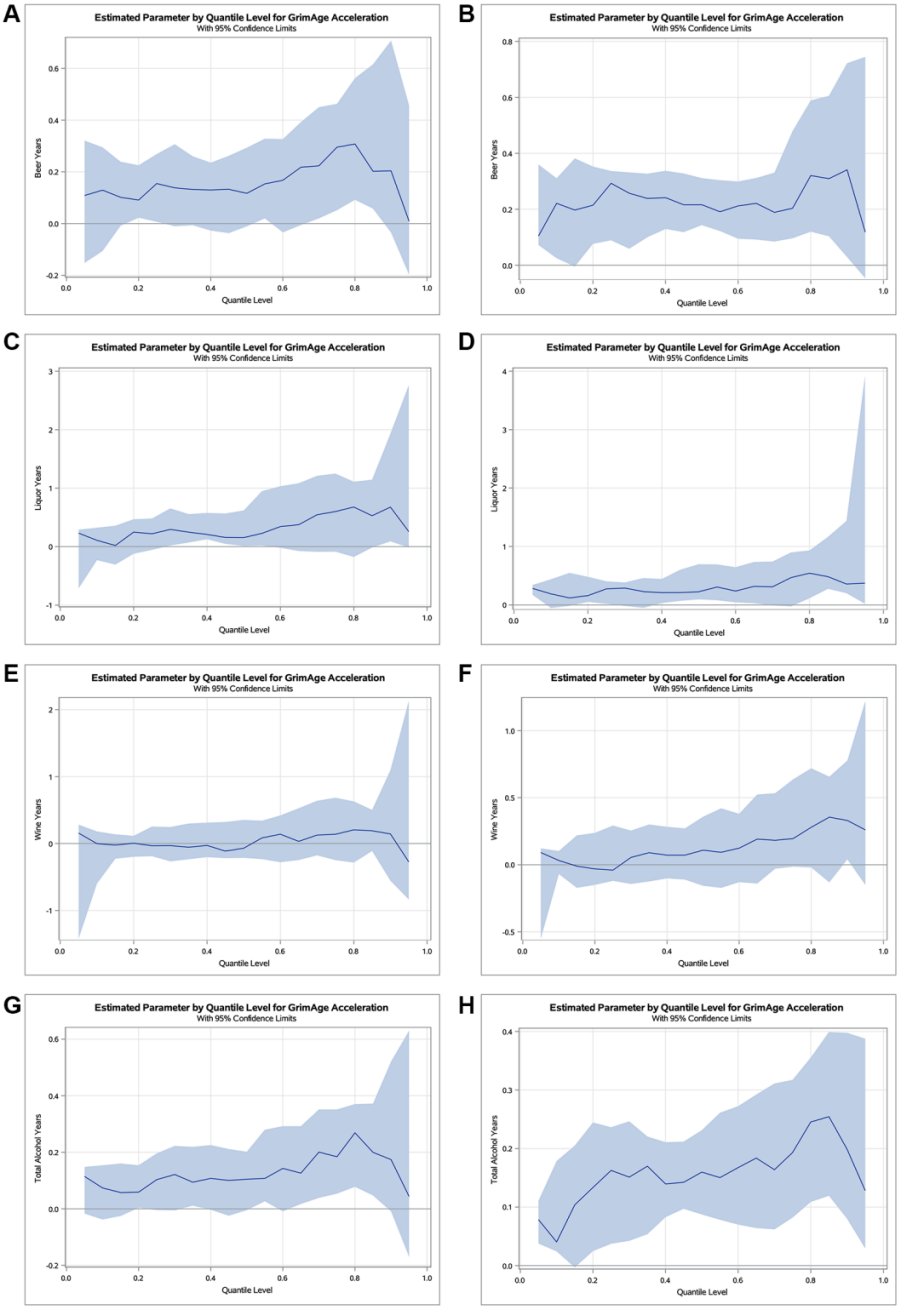
**Estimated parameters by quantile with 95% confidence limits for the effect of cumulative alcohol consumption on GAA at examination years 15 and 20.** Quantile regression plots for beer (**A**, **B**), liquor (**C**, **D**), wine (**E**, **F**), and total alcohol (**G**, **H**) years at Y15 and Y20, respectively. The x-axis represents the quantile scale, and the y-axis represents the effect of alcohol on GAA for a given quantile. Results are adjusted for chronological age, sex, race, center, education, pack years of smoking, BMI, and physical activity.

Figure 1 displays quantile regression plots for the cumulative alcohol consumption variables at Y15 and Y20 on GAA. We plotted regression estimates for 19 quantiles ranging from 0.05 to 0.95. As displayed in the plots, beer years displayed an increasing effect on GAA across the distribution at Y15 with almost a three and a half times greater effect in the upper conditional quartiles compared to the lower conditional quartiles (0.31-year vs. 0.09-year, respectively; Figure 1A), with similar effects at Y20 (0.34-year vs. 0.10-year, respectively; Figure 1B). Liquor years demonstrated a thirty-four times greater effect in the upper conditional quartiles compared to the lower conditional quartiles at Y15 (0.68-year vs. 0.02-year, respectively; Figure 1C) and five times greater effect in the upper conditional quartiles compared to the lower conditional quartiles at Y20 (0.54-year vs. 0.11-year, respectively; Figure 1D). Wine years displayed a relatively negative effect on GAA in the lower conditional quartiles with a positive effect in the upper conditional quartiles at both Y15 and Y20. Notably, the effect estimate of wine years can be six and a half times greater in the upper conditional quartiles compared to the lower conditional quartiles of the distribution at Y15 (0.20-year gain vs. 0.03-year loss, respectively; Figure 1E) and nine times greater effect in the upper conditional quartiles compared to the lower conditional quartiles at Y20 (0.36-year gain vs. 0.04-year loss, respectively; Figure 1F). Total alcohol years demonstrated a four and a half times greater effect in the upper conditional quartiles of the distribution compared to the lower conditional quartiles (0.27-year vs. 0.06-year, respectively; Figure 1G) at Y15 and an approximately six times greater effect in the upper conditional quartiles compared to the lower conditional quartiles (0.25-year vs. 0.04-year, respectively; Figure 1H) at Y20.

### Recent binge drinking on epigenetic age acceleration

Table 3 presents results for the associations between binge drinking and EAA. Recent binge drinking was positively associated with GAA at Y15 (*P* < 0.001) and Y20 (*P* < 0.001). Compared to participants who did not binge drink in the past 30 days, those who did exhibited a 1.22-year [95% CI: 0.69, 1.76] and a 1.55-year [95% CI: 0.99, 2.11] higher GAA at Y15 and Y20, respectively. Additionally, the number of days of binge drinking was associated with GAA at Y15 (*P* = 0.002) and Y20 (*P* < 0.001). Each additional day of binge drinking was associated with a 0.11-year [95% CI: 0.04, 0.18] and a 0.21-year [95% CI: 0.12, 0.29] higher GAA at Y15 and Y20, respectively. GEE yielded comparable associations as Y15 and Y20. IEAA, EEAA, and PAA were not associated with either binge drinking variable. Restricting to study participants with complete follow up yielded consistent conclusions (Supplementary Table 8). We observed similar correlations between days of recent binge drinking and several GrimAge surrogate biomarkers of blood plasma proteins at Y15 and Y20 (Supplementary Figures 1, 2). When modeling both cumulative alcohol consumption and recent binge drinking during GAA analyses, we observed recent binge drinking remained associated with GAA, while the cumulative alcohol consumption variables became less significant, although liquor years remained associated with GAA (Supplementary Table 9). Recent binge drinking was associated with telomere length derived from DNA methylation (Supplementary Table 7).

**Table 3:** Analysis results for the association between recent binge drinking and EAA at examination years 15 and 20.

	**Year 15**		**Year 20**		**GEE**	
	**β [95% CI]**	***P***	**β [95% CI]**	***P***	**β [95% CI]**	***P***
Recent Binge						
IEAA	–0.33 [–0.97, 0.32]	0.320	–0.30 [–0.99, 0.38]	0.386	–0.32 [–0.80, 0.16]	0.195
EEAA	–0.31 [–1.08, 0.46]	0.429	–0.60 [–1.36, 0.15]	0.115	–0.45 [–1.02, 0.13]	0.126
PAA	0.22 [–0.67, 1.12]	0.624	0.27 [–0.65, 1.20]	0.563	0.24 [–0.44, 0.93]	0.486
GAA	1.22 [0.69, 1.76]	<0.001	1.55 [0.99, 2.11]	<0.001	1.38 [0.92, 1.83]	<0.001
Recent Binge Quantity						
IEAA	–0.04 [–0.12, 0.05]	0.389	0.00 [–0.10, 0.10]	0.983	–0.02 [–0.12, 0.08]	0.654
EEAA	–0.06 [–0.16, 0.04]	0.244	0.03 [–0.08, 0.15]	0.575	–0.02 [–0.10, 0.06]	0.635
PAA	–0.04 [–0.16, 0.07]	0.475	0.14 [0.00, 0.28]	0.055	0.03 [–0.07, 0.13]	0.579
GAA	0.11 [0.04, 0.18]	0.002	0.21 [0.12, 0.29]	<0.001	0.15 [0.07, 0.23]	<0.001

Results are adjusted for chronological age, sex, race, center, education, pack years of smoking, BMI, and physical activity. Beta coefficients for recent binge represents the gain in EAA for participants who binge drank in the past 30 days and the beta coefficients for recent binge quantity represents the gain in EAA for each additional day of binge drinking in the past 30 days. Abbreviations: IEAA: intrinsic epigenetic age acceleration; EEAA: extrinsic epigenetic age acceleration; PAA: PhenoAge acceleration; GAA: GrimAge acceleration.

### Cumulative alcohol consumption and chronological age interaction on GrimAge acceleration

Table 4 presents the interaction and stratified results for the joint associations of cumulative alcohol consumption and chronological age on GAA. Summary statistics by age quartiles are presented in Supplementary Table 10. At Y15, we observed a 0.35-year [95% CI: 0.14, 0.57] higher GAA per 5 beer years among participants in quartile 1 compared to a 0.05-year [95% CI: –0.30, 0.19] loss in GAA among those in quartile 4 (*P*_interaction_ < 0.001). Additionally, participants in quartile 1 displayed a 0.26-year [95% CI: 0.11, 0.41] higher GAA per 5 total alcohol years compared to participants in quartile 4 who experienced a 0.06-year [95% CI: –0.11, 0.22] higher GAA (*P*_interaction_ = 0.002). No statistical interactions were observed between liquor and wine years with chronological age.

**Table 4:** Interaction and stratified analysis results for the association between cumulative alcohol consumption and GAA at examination years 15 and 20 by strata of chronological age quartiles.

	**Year 15**		**Year 20**		**GEE**	
	**Β_alcohol_ [95% CI]**	***P***	**Β_alcohol_ [95% CI]**	***P***	**Β_alcohol_ [95% CI]**	***P***
Beer Years	**–0.05 [–0.07, –0.02]**	<0.001^*^	**–0.04 [–0.06, –0.03]**	<0.001^*^	**–0.03 [–0.05, –0.02]**	<0.001^*^
Quartile 1	0.35 [0.14, 0.57]	0.002	0.29 [0.12, 0.46]	<0.001	0.31 [0.16, 0.46]	<0.001
Quartile 2	0.30 [0.10, 0.50]	0.004	0.36 [0.15, 0.57]	<0.001	0.32 [0.15, 0.49]	<0.001
Quartile 3	–0.07 [–0.28, 0.13]	0.488	0.29 [0.13, 0.45]	<0.001	0.13 [–0.03, 0.28]	0.119
Quartile 4	–0.05 [–0.30, 0.19]	0.660	–0.17 [–0.34, –0.01]	0.043	–0.16 [–0.34, 0.03]	0.094
Liquor Years	**–0.03 [–0.08, 0.02]**	**0.297**	**–0.01 [–0.05, 0.04]**	**0.766**	**–0.02 [–0.06, 0.03]**	**0.500**
Quartile 1	0.50 [0.14, 0.87]	0.007	0.22 [–0.05, 0.50]	0.115	0.32 [–0.01, 0.65]	0.060
Quartile 2	0.18 [–0.29, 0.66]	0.440	0.21 [–0.25, 0.67]	0.363	0.18 [–0.25, 0.60]	0.416
Quartile 3	0.00 [–0.51, 0.51]	0.999	0.43 [0.08, 0.78]	0.017	0.23 [–0.19, 0.65]	0.278
Quartile 4	0.39 [–0.01, 0.79]	0.055	0.26 [–0.11, 0.63]	0.164	0.31 [–0.07, 0.70]	0.114
Wine Years	**–0.04 [–0.12, 0.04]**	**0.312**	**–0.07 [–0.13, –0.01]**	0.015^*^	**–0.04 [–0.09, 0.01]**	**0.154**
Quartile 1	0.46 [–0.44, 1.37]	0.317	0.64 [–0.07, 1.34]	0.077	0.54 [–0.08, 1.16]	0.090
Quartile 2	–0.07 [–0.57, 0.43]	0.777	0.05 [–0.29, 0.39]	0.784	0.00 [–0.27, 0.26]	0.991
Quartile 3	–0.03 [–0.52, 0.46]	0.908	0.23 [–0.07, 0.53]	0.129	0.12 [–0.10, 0.34]	0.290
Quartile 4	0.12 [–0.31, 0.56]	0.579	–0.17 [–0.48, 0.15]	0.297	–0.08 [–0.45, 0.30]	0.690
Total Alcohol Years	**–0.03 [–0.05, –0.01]**	0.002^*^	**–0.03 [–0.04, –0.02]**	<0.001^*^	**–0.02 [–0.04, –0.01]**	0.004^*^
Quartile 1	0.26 [0.11, 0.41]	<0.001	0.20 [0.08, 0.31]	0.001	0.22 [0.10, 0.33]	<0.001
Quartile 2	0.18 [0.03, 0.34]	0.019	0.23 [0.08, 0.38]	0.004	0.19 [0.06, 0.33]	0.006
Quartile 3	–0.05 [–0.21, 0.12]	0.558	0.23 [0.12, 0.35]	<0.001	0.11 [0.00, 0.22]	0.045
Quartile 4	0.06 [–0.11, 0.22]	0.497	–0.07 [–0.19, 0.04]	0.192	–0.05 [–0.20, 0.11]	0.550

^*^Interaction terms with *P* ≤ 0.05. Bolded values represent the beta coefficient [95% CI] and *P* for the joint association between alcohol years and chronological age. Results are adjusted for sex, race, center, education, pack years of smoking, BMI, and physical activity. Beta coefficients represent the gain in EAA for each additional 5 alcohol years. Quartile 1: Y15_[32≤age≤37]_, Y20_[37≤age≤42]_; Quartile 2: Y15_[38≤age≤40]_, Y20_[43≤age≤45]_; Quartile 3: Y15_[41≤age≤43]_, Y20_[46≤age≤48]_; and Quartile 4: Y15_[44≤age≤49]_, Y20_[49≤age≤54]_.

At Y20, we observed a 0.29-year [95% CI: 0.12, 0.46] higher GAA in quartile 1 per 5 beer years compared to a 0.17-year [95% CI: –0.34, –0.01] loss in GAA in quartile 4 (*P*_interaction_ < 0.001). Moreover, quartile 1 experienced a 0.20-year [95% CI: 0.08, 0.31] higher GAA compared to a 0.07-year [95% CI: –0.19, 0.04] loss in GAA in quartile 4 per 5 total alcohol years (*P*_interaction_ < 0.001). Interaction and stratified results from GEE provided similar findings. Restricting to study participants with complete follow up yielded consistent conclusions (Supplementary Table 11). We observed similar results when adjusting for both cumulative alcohol consumption and recent binge drinking (Supplementary Table 12). While interactions of cumulative alcohol consumption with sex yielded primarily non-significant associations, female participants displayed higher GAA with greater consumption of beer, wine, and total alcohol compared to male participants (Supplementary Table 13). Additionally, Black participants experienced a higher GAA with greater consumption of liquor and total alcohol compared to White participants, although these interactions were non-significant (Supplementary Table 14).

## DISCUSSION

We observed positive associations between cumulative alcohol consumption and binge drinking with GAA in young adults. Specifically, we observed positive associations between liquor and total alcohol years with GAA. Beer and wine years were marginally and not associated with GAA, respectively. We also observed positive associations between recent binge drinking and each additional day of recent binge drinking with GAA, respectively. Additionally, we identified statistical interactions between beer and total alcohol years with chronological age, with younger participants exhibiting a higher average in GAA compared to older participants. Our results provide novel insight into the association of cumulative and recent alcohol consumption, as well as the type of alcohol, on age-related epigenetic changes.

Estimated biological age via DNA methylation has provided unique molecular insights into the aging process and age-related conditions. Additionally, these metrics have furthered our understanding of the impact comorbidities, behaviors, and lifestyle factors, including alcohol consumption, have on epigenetic age-related changes. We observed cumulative alcohol consumption was positively associated with GAA across time, suggesting long-term alcohol consumption may accelerate epigenetic aging. Alcohol has previously been associated with several of the GrimAge surrogate biomarkers of blood plasma proteins, including PAI-1 [20] and leptin [21]. We observed similar correlations between these surrogate biomarkers of blood plasma proteins and several alcohol variables at Y15 and Y20, suggesting the observed associations between alcohol consumption and epigenetic aging as estimated by GAA may be partially explained by the correlations between alcohol and the surrogate biomarkers of blood plasma proteins of GrimAge. Our findings, i.e., positive association between total alcohol consumption and epigenetic aging, are consistent with previously observed associations [11–16]. Findings from our study add to the current literature by identifying associations between cumulative, lifetime alcohol consumption and epigenetic age acceleration in a diverse study population, as well as showing increasing effects on epigenetic aging with higher alcohol consumption, as demonstrated during the quantile regression analyses. Our findings, together with previous studies, illustrate the GrimAge surrogate biomarkers of blood plasma proteins may be modulated by alcohol consumption, with potential subsequent negative impacts on the aging process. Behavioral modifications to limit alcohol consumption may reduce alcohol induced epigenetic age-related changes and potentially, increase lifespan.

The type of alcohol consumed has been shown to have varying associations with numerous age-related diseases [4, 22, 23]. In our study, we observed positive associations between beer, liquor, and total alcohol years with GAA and notably, null associations with wine years. Prior studies have investigated the molecular and cellular mechanisms underlying the varying effects of alcohol consumption on the aging process. Polyphenols, a group of naturally occurring anti-oxidants, have been suggested to modulate adaptive immune responses and influence anti-aging mechanisms, including preventing cellular senescence, and are present in alcohol by various quantities [24]. The hops and malt in beer contain phenolic compounds that exhibit anti-carcinogenic and anti-inflammatory properties, such as inhibiting inducible nitric oxide (NO) synthase and cyclooxygenase 2 (COX2) [25]. Production of NO and COX2 have been associated with cellular aging and may modulate epigenetic aging through similar mechanisms [26, 27]. Additionally, moderate beer consumption has been associated with lower risk of cardiovascular disease, potentially in part by the cardioprotective effects of polyphenols [28, 29]. Contrary to these findings, our results showed accelerated epigenetic aging with beer consumption, demonstrating potential independent associations between age-related epigenetic changes and disease. Compared to beer and wine, liquor has the highest alcohol content but the lowest polyphenolic concentration, which may impact the beneficial health gains as seen with wine and beer [25]. Liquor has been associated with greater risk of lung [4], and upper digestive tract cancers [30] and overall mortality [31], suggesting potentially negative impacts of liquor on the aging process and age related diseases. Wine contains resveratrol, a polyphenol that exhibits anti-aging properties by acting as a potent SIRT1 activator to regulate longevity and has shown to improve health and longevity in mice models [32]. As a part of the Mediterranean diet, moderate wine consumption has been shown to protect against age-related diseases including cancer [4, 33] and dementia [34] and increase life expectancy [35]. While we observed associations with alcohol consumption and GAA, the null findings with the other EAA metrics may be partially explained by different sets of CpGs and covariates used in the development of the epigenetic age measures, which captures different biological processes of aging. While previous studies examined total alcohol consumption and EAA [11–16], we identified associations with cumulative alcohol specific consumption and epigenetic aging, providing novel insight into alcohol specific effects on the epigenome. In sum, these findings suggest the type of alcohol may influence epigenetic age-related changes differently and as such, altering beverage preferences may modulate the aging process.

Alcohol’s association on age-related epigenetic changes appear to differ by the pattern and severity of consumption. We observed large effect estimates for recent binge drinking on GAA, although the effect estimates for the number of days of binge drinking were similar to those observed for cumulative alcohol consumption. The large, although transient, effect of binge drinking on GAA may be due to pharmacokinetics, where the rate of absorption is greater than the rate of elimination during binging episodes, resulting in high blood alcohol concentrations. Compared to non-binge drinking consumption, binge drinking leads to acutely elevated levels of alcohol, which can negatively impact the body through organ injury, local and systemic inflammation, and endotoxemia, leading to potential multisystemic pathophysiological consequences [36]. Furthermore, the generation of reactive oxygen species and alcohol metabolites and disruption of anti-oxidant mechanisms, leads to cellular dysfunction and immune system dysfunction and exhaustion [36]. Binge drinking has also been associated with PAI-1 [37], suggesting the observed association between binge drinking and GAA may be partially explained by the correlations between binge drinking and the surrogate biomarkers of blood plasma proteins of GrimAge. Our results demonstrate differences in the effect of cumulative and binge alcohol consumption patterns on epigenetic aging, and suggests lifestyle changes, such as limiting binge drinking, may aid in slowing biological aging.

Consumption of alcohol varies with chronological age, with the prevalence and quantity inversely associated with age [5, 38]. While the mechanisms for the observed statistical interactions by chronological age remains unclear, one possible explanation involves age-related changes in alcohol preference. Findings from five national alcohol surveys showed beer and liquor consumption decline with increasing age, while wine consumption exhibited a more complex pattern [39]. In the current study, older participants exhibited greater wine years and lower beer and liquor years, suggesting that changes in alcohol consumption patterns may modulate age-related epigenetic changes. An additional explanation maybe due to alterations in the pattern of alcohol consumption. For example, binge drinking has previously been inversely associated with chronological age [40]. We observed participants in the highest age quartile reported the lowest binging events compared to other quartiles, consistent with declining excessive drinking with increasing age. Our findings suggest age-related changes to alcohol consumption may influence epigenetic aging and may serve to slow biological aging later in life.

As a longitudinal study with a large sample size, we were able to obtain multiple alcohol and DNA methylation measurements. Additionally, the detailed collection of type-specific alcohol consumption data allowed us to elucidate specific effects on EAA. This study, however, is not without limitations. Alcohol consumption was self-reported, which may be subject to social desirability bias in which participants report levels of consumption that are socially acceptable. However, consistent findings were observed with multiple time points and all responses at each examination were confidential. Despite being a diverse study population, CARDIA enrolled Black and White participants at four locations across the United States and as such, additional studies with more diverse populations are needed to better generalize these findings. Additionally, null associations between wine and the EAA metrics may be related to the sample size of this study and thus, replication of the cumulative alcohol specific associations in larger studies are needed to validate the findings presented here. Furthermore, race is a social construct and studies investigating genetic ancestry, as estimated by single nucleotide polymorphisms, may provide additional insights into the association between alcohol consumption and biological aging. Finally, due to the age of the study population, we were unable to link associations to any clinically significant, alcohol-related outcomes. Future research in older populations, and as CARDIA ages, will help elucidate the complete relationship between alcohol, epigenetics, and health.

In conclusion, we observed significant associations between cumulative and recent alcohol consumption, as well as the type of alcohol, with GAA. Moreover, we observed statistical interactions between cumulative alcohol consumption and chronological age on GAA. The findings presented here add to the existing literature on alcohol and the aging process and provide novel insights into the heterogeneous effects of the type of alcohol on epigenetic aging. Replication of our findings is needed as well as further exploration of the causal mechanisms of alcohol on epigenetic age-related changes. With the high prevalence of alcohol consumption and the growing aging population in the United States, elucidating changes to the epigenome due to alcohol consumption may yield novel insights into the aging process and potentially, longevity facilitating lifestyle modifications.

## METHODS

### Study sample

Details of the original CARDIA study design and examinations have previously been described [41]. Briefly, CARDIA is a population-based, cohort study designed to investigate the determinants of subclinical and clinical cardiovascular disease. Four centers across the United States recruited 5,115 Black and White participants ages 18 to 30 years between 1985 to 1986 and received in person examinations at 1985-1986 (baseline visit; year 0 [Y0]), 1987-88 (Y2), 1990-91 (Y5), 1992-93 (Y7), 1995-96 (Y10), 2000-01 (Y15), 2005-06 (Y20), 2010-11 (Y25), 2015-16 (Y30), and currently participating in 2020-21 (Y35).

### Alcohol consumption measurements

Alcohol consumption was assessed at Y0 and at each follow up examination. Participants were asked “Did you drink any alcoholic beverages in the past year?” and if answered yes, participants were asked “How many drinks of [alcohol type] do you usually have per week?” for beer (a 12-ounce glass, can, or bottle), liquor (a shot or 1.5-ounces), and wine (a 5-ounce glass). Binge drinking was also obtained by asking participants “During the past 30 days, on how many days did you have five or more drinks on the same occasion?” We considered six alcohol variables at Y15 and Y20: four continuous alcohol variables capturing cumulative consumption from Y0 to Y15 and from Y0 to Y20, i.e., beer, liquor, wine, and total alcohol (the sum of the individual alcohol types); one binary variable indicating whether a participant binge drank in the past 30 days at Y15 and Y20; and one continuous variable indicating the number of days a participant binge drank during those 30 days at Y15 and Y20. In order to calculate cumulative alcohol consumption from all available CARDIA data from Y0 to Y15 and Y20 separately, we assumed weekly consumption of alcohol measured at each CARDIA exam was consistent in-between study examinations, i.e., weekly alcohol consumption represented consumption throughout the year and between examinations. For each weekly alcohol variable, we estimated ‘alcohol years’ by multiplying each alcohol variable by 52 weeks and the number of years between visits and dividing by 365 days, where an alcohol year is equivalent to 365 days of alcohol consumption. We then estimated alcohol years for each alcohol type separately and total alcohol by summing across examinations from Y0 to Y15 and Y20.

### DNA methylation profiling

Details of methylation profiling and quality control have previously been described [19, 42–44]. Briefly, a randomly selected subset of 1,200 study participants with available whole blood repeatedly collected at both Y15 and Y20 (a total of 2,400 samples) underwent DNA methylation profiling using the Illumina MethylationEPIC Beadchip. The R package ENmix was used to perform data preprocessing and quality control using default parameter settings [45]. Low quality methylation measurements were defined as having less than 3 beads or a detection *P* < 1 × 10^−6^. A total of 87 samples with low quality methylation measurements >5% or bisulfite conversion process of extremely low intensity (defined as less than 3 × standard deviation of the intensity across samples below the mean intensity) and 6,209 CpG sites with a detection rate <95% were excluded from further analysis. Furthermore, 95 samples were identified as extreme outliers via the average total intensity value [intensity of the unmethylated signal (*U*) + intensity of the methylated signal (*M*)] or *β* value [*M*/(*U* + *M* + 100)] across all CpG sites and Tukey’s method [46]. Application of a model-based background correction was conducted using ENmix and dye bias correction was performed using RELIC [47]. *M* and *U* intensities for Infinium I or II probes were quantile-normalized separately, respectively. Low quality methylation measurements and *β* value outliers were set to missing. After applying these quality control measures, the final methylation dataset for epigenetic age calculation included 1,042 and 957 samples at Y15 and Y20, respectively. Supplementary Figure 3 presents a graphical timeline of the examinations with phenotype and blood draw collection.

### Epigenetic age calculation

Four epigenetic ages were calculated at Y15 and Y20. Horvath’s age, intrinsic epigenetic age acceleration (IEAA), was calculated with 353 CpGs and is associated with cell-intrinsic aging [7]. Hannum’s age, extrinsic epigenetic age acceleration (EEAA), was estimated from 71 CpGs and is associated with immunological aging [8]. Levine’s age, PhenoAge acceleration (PAA), was calculated with 513 CpGs and is associated with comorbidities and physical functionality [9]. Lu’s age, GrimAge acceleration (GAA), was estimated from 1,030 CpGs and is associated with lifespan [10]. A publicly available online calculator was used to calculate the DNA-methylation epigenetic age estimates (https://dnamage.genetics.ucla.edu/new). EAA for each metric was estimated as the residuals from a linear model of chronological age and the top eight technical principal components, capturing technical biases and batch effect, on epigenetic age as previously described [42].

### Statistical analysis

Multiple linear regression and generalized estimating equations (GEE) were used to examine the associations between the cumulative and binge drinking alcohol variables (independent variables) and each EAA (outcome variables) at Y15 and Y20. Each cumulative alcohol variable was rescaled to units of five drink years (i.e., divided by 5) to aid in the interpretation of beta coefficients. Drinkers were further categorized into low, intermediate, or high consumption via tertiles and compared to non-drinkers for each cumulative alcohol variable. Quantile regression was additionally performed to evaluate the conditional quantiles of each cumulative alcohol variable on EAA. This approach provides a more comprehensive view of the covariate effects on variables under study [48]. Due to previously observed differences in alcohol consumption by demographic characteristics [5, 6], interaction and stratified analyses were conducted by chronological age, sex, and race with the cumulative alcohol variables. Age was categorized into quartiles during stratified analyses: quartile 1 (n_Y15[32≤age≤37]_ = 242, n_Y20[37≤age≤42]_ = 223), quartile 2 (n_Y15[38≤age≤40]_ = 246, n_Y20[43≤age≤45]_ = 224), quartile 3 (n_Y15[41≤age≤43]_ = 294, n_Y20[46≤age≤48]_ = 271), and quartile 4 (n_Y15[44≤age≤49]_ = 248, n_Y20[49≤age≤54]_ = 227). Sensitivity analyses using study participants with complete follow up were performed. All models were adjusted for chronological age, sex, race, center, education, pack years of smoking, body mass index, and physical activity. Pack years of smoking was estimated by converting the number of daily smoked cigarettes measured at each examination to packs of cigarettes, multiplying by the number of years between examinations, and summing the products from Y0 to Y15 and Y20, separately. Associations were declared significant if *P* ≤ 0.05. All statistical analyses were performed using SAS 9.4.

## Supplementary Materials

Supplementary Figures

Supplementary Tables
