# Supplementary material for: Alcohol consumption and epigenetic age acceleration in young adults
Source: Aging (Albany NY). 2023 Jan 5;15(2):371–95. doi: 10.18632/aging.204467 (PMC9925681; doi:10.18632/aging.204467)
Supplement: Supplementary Figures [file aging-15-204467-s001.pdf]

SUPPLEMENTARY FIGURES

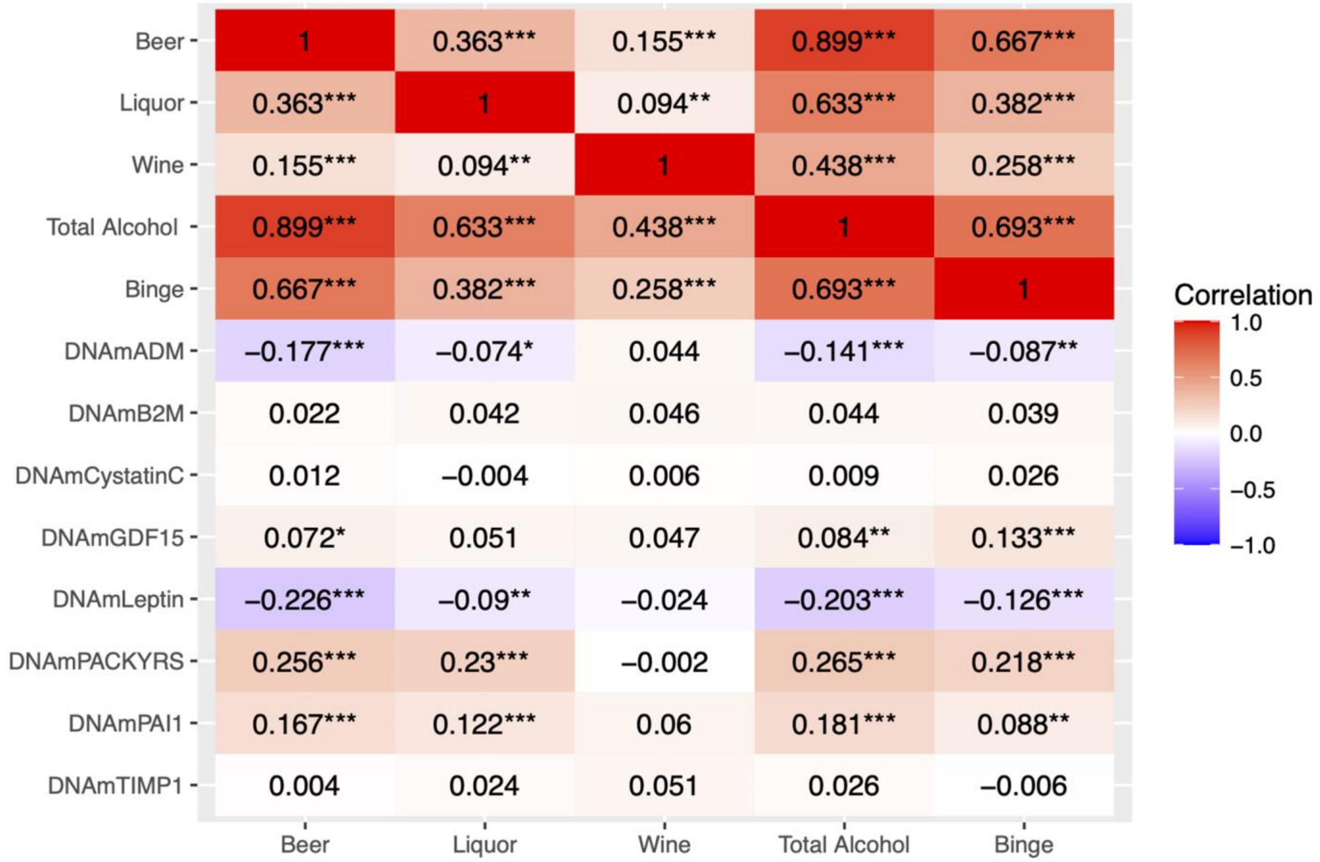

	Beer	Liquor	Wine	Total Alcohol	Binge
Beer	1	0.363***	0.155***	0.899***	0.667***
Liquor	0.363***	1	0.094**	0.633***	0.382***
Wine	0.155***	0.094**	1	0.438***	0.258***
Total Alcohol	0.899***	0.633***	0.438***	1	0.693***
Binge	0.667***	0.382***	0.258***	0.693***	1
DNAmADM	−0.177***	−0.074*	0.044	−0.141***	−0.087**
DNAmB2M	0.022	0.042	0.046	0.044	0.039
DNAmCystatinC	0.012	−0.004	0.006	0.009	0.026
DNAmGDF15	0.072*	0.051	0.047	0.084**	0.133***
DNAmLeptin	−0.226***	−0.09**	−0.024	−0.203***	−0.126***
DNAmPACKYRS	0.256***	0.23***	−0.002	0.265***	0.218***
DNAmPAI1	0.167***	0.122***	0.06	0.181***	0.088**
DNAmTIMP1	0.004	0.024	0.051	0.026	−0.006

Supplementary Figure 1. Pairwise correlation of cumulative alcohol consumption and binge drinking and DNA methylation-based biomarkers of GrimAge at Y15. The columns represent the cumulative alcohol consumption variables and the number of days of binge drinking and the rows represent the DNA methylation-based biomarkers of GrimAge with the Pearson correlation coefficient reported within each box. $^{***}P < 0.001$, $^{**}P < 0.01$, $^{*}P < 0.05$. Abbrevations: DNAmADM: adrenomedullin; DNAmB2M: beta-2 microglobulin; DNAmCystatinC: cystatin C; DNAmGDF15: growth differentiation factor 15; DNAmLeptin: leptin; DNAmPACKYRS: smoking pack-years; DNAmPAI1: plasminogen activation inhibitor 1; DNAmTIMP1: tissue inhibitor metalloproteinase 1.

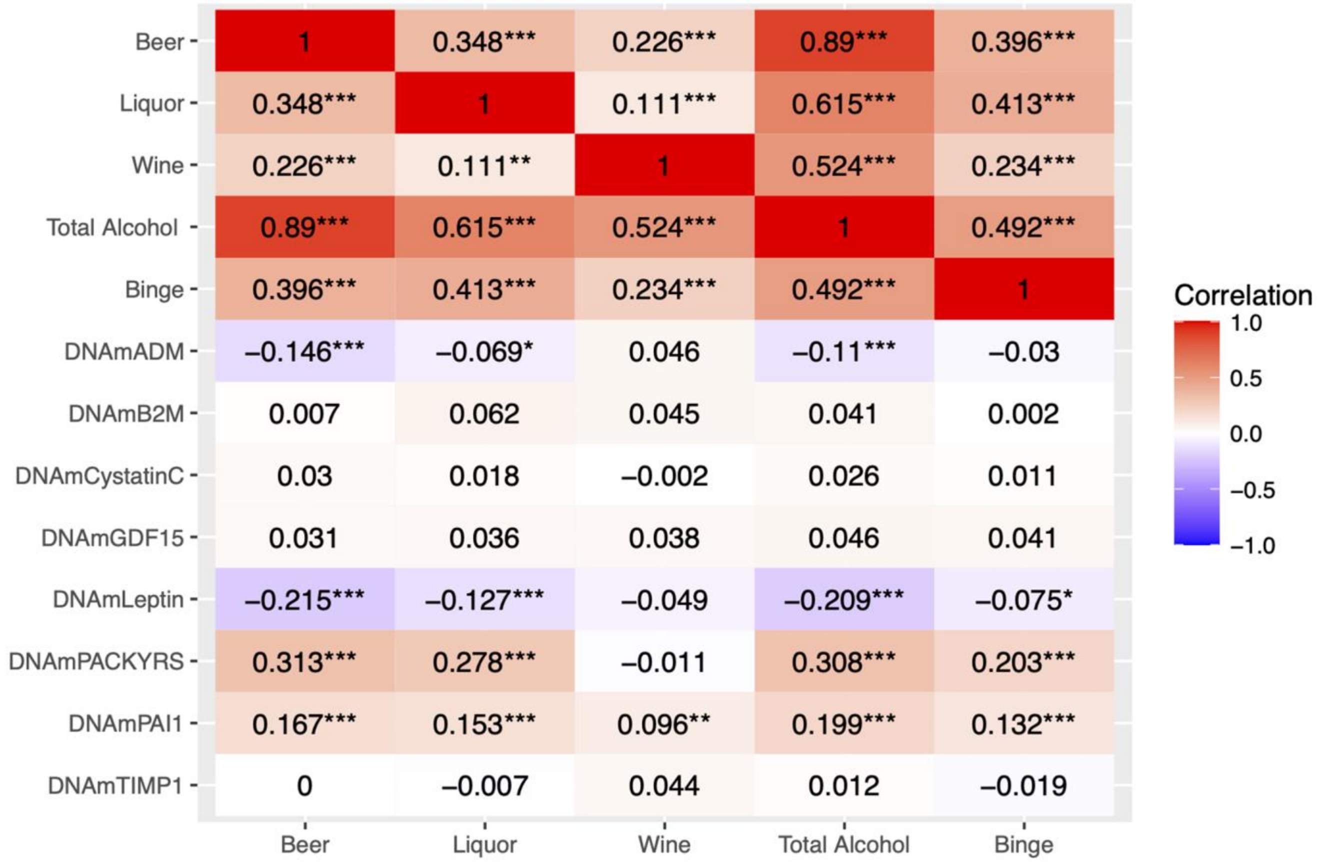

Supplementary Figure 2. Pairwise correlation of cumulative alcohol consumption and binge drinking and DNA methylation-based biomarkers of GrimAge at Y20. The columns represent the cumulative alcohol consumption variables and the number of days of binge drinking and the rows represent the DNA methylation-based biomarkers of GrimAge with the Pearson correlation coefficient reported within each box. $^{***}P < 0.001$, $^{**}P < 0.01$, $^{*}P < 0.05$. Abbrevations: DNAmADM: adrenomedullin; DNAmB2M: beta-2 microglobulin; DNAmCystatinC: cystatin C; DNAmGDF15: growth differentiation factor 15; DNAmLeptin: leptin; DNAmPACKYRS: smoking pack-years; DNAmPAI1: plasminogen activation inhibitor 1; DNAmTIMP1: tissue inhibitor metalloproteinase 1.

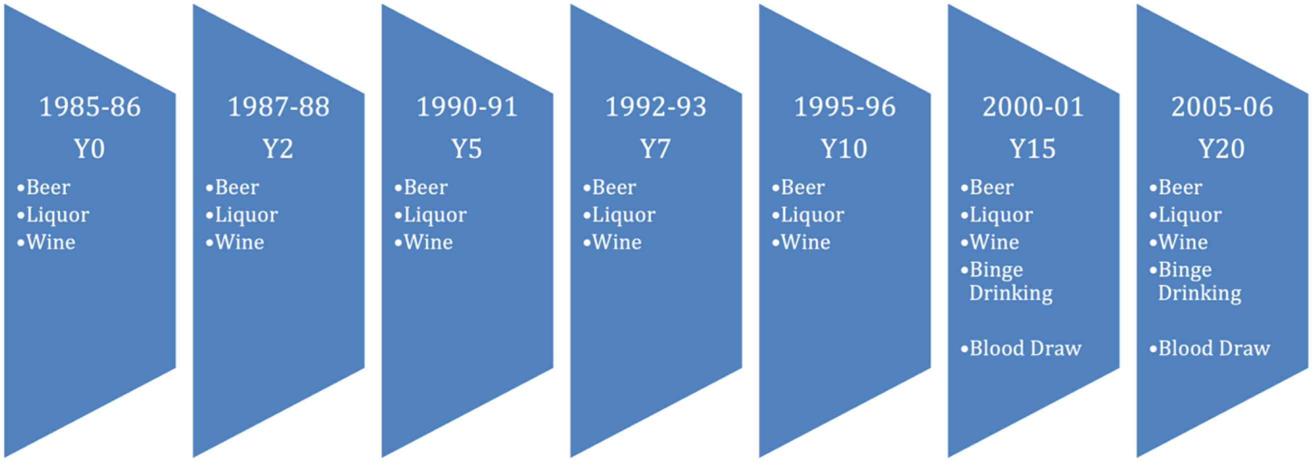

Supplementary Figure 3. CARDIA study timeline and data collection. Chronological timeline of alcohol consumption and blood collection in the CARDIA study.
