# Supplementary material for: Alcohol consumption and epigenetic age acceleration in young adults
Source: Aging (Albany NY). 2023 Jan 5;15(2):371–95. doi: 10.18632/aging.204467 (PMC9925681; doi:10.18632/aging.204467)
Supplement: Supplementary Tables [file aging-15-204467-s002.pdf]

SUPPLEMENTARY TABLES

Supplementary Table 1. Analysis results for the association between cumulative alcohol consumption and EAA at examination years 15 and 20 among participants with complete follow-up.

	Year 15		Year 20		GEE	
	β [95% CI]	*P*	β [95% CI]	*P*	β [95% CI]	*P*
Beer Years						
IEAA	–0.02 [–0.16, 0.11]	0.730	–0.09 [–0.20, 0.02]	0.125	–0.06 [–0.17, 0.05]	0.274
EEAA	0.01 [–0.15, 0.17]	0.892	–0.04 [–0.16, 0.09]	0.566	–0.02 [–0.13, 0.10]	0.796
PAA	0.14 [–0.04, 0.33]	0.126	0.01 [–0.14, 0.16]	0.934	0.06 [–0.07, 0.18]	0.355
GAA	0.11 [0.00, 0.22]	0.050	0.13 [0.04, 0.22]	0.004	0.11 [–0.03, 0.26]	0.125
Liquor Years						
IEAA	0.16 [–0.12, 0.43]	0.273	–0.12 [–0.34, 0.10]	0.281	–0.02 [–0.24, 0.20]	0.850
EEAA	–0.10 [–0.43, 0.23]	0.548	–0.04 [–0.29, 0.20]	0.734	–0.06 [–0.25, 0.12]	0.506
PAA	0.33 [–0.05, 0.71]	0.093	0.12 [–0.18, 0.42]	0.442	0.19 [–0.11, 0.49]	0.204
GAA	0.29 [0.06, 0.52]	0.015	0.29 [0.11, 0.47]	0.001	0.27 [0.07, 0.47]	0.008
Wine Years						
IEAA	0.32 [–0.01, 0.66]	0.059	–0.02 [–0.25, 0.22]	0.899	0.10 [–0.07, 0.27]	0.270
EEAA	0.02 [–0.38, 0.42]	0.936	–0.14 [–0.40, 0.12]	0.286	–0.09 [–0.33, 0.16]	0.476
PAA	0.34 [–0.12, 0.80]	0.152	–0.05 [–0.37, 0.27]	0.747	0.07 [–0.25, 0.38]	0.685
GAA	0.06 [–0.22, 0.34]	0.665	0.06 [–0.13, 0.25]	0.544	0.06 [–0.16, 0.27]	0.620
Total Alcohol Years						
IEAA	0.03 [–0.06, 0.13]	0.493	–0.06 [–0.14, 0.02]	0.132	–0.02 [–0.10, 0.05]	0.515
EEAA	–0.01 [–0.12, 0.11]	0.928	–0.04 [–0.12, 0.05]	0.380	–0.02 [–0.10, 0.05]	0.541
PAA	0.15 [0.01, 0.28]	0.033	0.01 [–0.09, 0.12]	0.826	0.06 [–0.04, 0.16]	0.220
GAA	0.10 [0.02, 0.18]	0.016	0.11 [0.04, 0.17]	0.001	0.10 [0.00, 0.19]	0.043

Results are adjusted for chronological age, sex, race, center, education, pack years of smoking, BMI, and physical activity. Beta coefficients represent the gain in EAA for each additional 5 alcohol years. Abbreviations: IEAA: intrinsic epigenetic age acceleration; EEAA: extrinsic epigenetic age acceleration; PAA: PhenoAge acceleration; GAA: GrimAge acceleration. Number of participants with complete follow up: $n_{Y15} = 890$; $n_{Y20} = 820$.

Supplementary Table 2. Analysis results for the association between categorized alcohol consumption and EAA at examination year 15.

	Non-drinkers		Low		Intermediate		High	
	β [95% CI]	P_{trend}	β [95% CI]	*P*	β [95% CI]	*P*	β [95% CI]	*P*
Beer Years								
IEAA	Reference	0.742	–0.38 [–1.11, 0.35]	0.305	0.26 [–0.50, 1.02]	0.504	–0.34 [–1.15, 0.47]	0.408
EEAA	Reference	0.367	–0.77 [–1.64, 0.10]	0.083	–0.09 [–1.00, 0.81]	0.838	–0.63 [–1.60, 0.34]	0.200
PAA	Reference	0.539	–1.01 [–2.02, 0.01]	0.051	0.16 [–0.90, 1.21]	0.771	0.14 [–0.98, 1.27]	0.805
GAA	Reference	<0.001	0.45 [–0.16, 1.05]	0.146	0.66 [0.03, 1.29]	0.041	1.61 [0.94, 2.29]	<0.001
Liquor Years								
IEAA	Reference	0.764	–0.34 [–1.06, 0.37]	0.349	–0.19 [–0.94, 0.56]	0.616	0.20 [–0.54, 0.95]	0.596
EEAA	Reference	0.953	–0.37 [–1.22, 0.49]	0.401	–0.23 [–1.13, 0.66]	0.608	0.05 [–0.84, 0.94]	0.912
PAA	Reference	0.030	0.11 [–0.88, 1.10]	0.831	0.67 [–0.37, 1.71]	0.207	1.09 [0.06, 2.12]	0.039
GAA	Reference	<0.001	0.44 [–0.16, 1.03]	0.147	0.62 [–0.01, 1.24]	0.053	1.25 [0.63, 1.87]	<0.001
Wine Years								
IEAA	Reference	0.793	–0.67 [–1.40, 0.05]	0.069	0.20 [–0.53, 0.93]	0.589	–0.27 [–1.05, 0.50]	0.488
EEAA	Reference	0.046	–1.12 [–1.99, –0.26]	0.011	–0.64 [–1.51, 0.24]	0.153	–0.93 [–1.85, –0.01]	0.048
PAA	Reference	0.516	–0.65 [–1.65, 0.36]	0.208	0.02 [–1.00, 1.04]	0.970	–0.49 [–1.57, 0.58]	0.369
GAA	Reference	0.233	0.02 [–0.58, 0.63]	0.940	0.13 [–0.49, 0.74]	0.682	0.42 [–0.23, 1.07]	0.204
Total Alcohol Years								
IEAA	Reference	0.882	–0.25 [–1.06, 0.57]	0.550	0.16 [–0.67, 0.99]	0.702	–0.10 [–0.97, 0.78]	0.829
EEAA	Reference	0.351	–0.17 [–1.14, 0.80]	0.726	–0.16 [–1.15, 0.82]	0.744	–0.51 [–1.55, 0.52]	0.332
PAA	Reference	0.096	0.27 [–0.86, 1.40]	0.640	0.68 [–0.47, 1.83]	0.246	0.93 [–0.28, 2.14]	0.131
GAA	Reference	<0.001	0.49 [–0.19, 1.16]	0.155	0.62 [–0.07, 1.30]	0.077	1.57 [0.85, 2.29]	<0.001

Results are adjusted for chronological age, sex, race, center, education, pack years of smoking, BMI, and physical activity. Beta coefficients represent the gain in EAA for each alcohol category compared to non-drinkers. Abbreviations: IEAA: intrinsic epigenetic age acceleration; EEAA: extrinsic epigenetic age acceleration; PAA: PhenoAge acceleration; GAA: GrimAge acceleration.

Supplementary Table 3. Analysis results for the association between categorized alcohol consumption and EAA at examination year 20.

	Non-drinkers		Low		Intermediate		High	
	β [95% CI]	P_{trend}	β [95% CI]	*P*	β [95% CI]	*P*	β [95% CI]	*P*
Beer Years								
IEAA	Reference	0.020	–0.44 [–1.22, 0.34]	0.270	–0.40 [–1.22, 0.43]	0.345	–1.13 [–2.00, –0.25]	0.012
EEAA	Reference	0.123	–0.62 [–1.47, 0.24]	0.158	–1.18 [–2.07, –0.28]	0.010	–0.51 [–1.46, 0.45]	0.296
PAA	Reference	0.478	–0.66 [–1.72, 0.39]	0.217	–0.35 [–1.46, 0.75]	0.531	0.51 [–0.66, 1.69]	0.394
GAA	Reference	<0.001	0.44 [–0.20, 1.07]	0.176	0.53 [–0.14, 1.19]	0.119	2.05 [1.35, 2.76]	<0.001
Liquor Years								
IEAA	Reference	0.031	0.22 [–0.57, 1.01]	0.583	–0.73 [–1.51, 0.05]	0.068	–0.73 [–1.56, 0.09]	0.081
EEAA	Reference	0.084	–0.45 [–1.31, 0.42]	0.311	–1.09 [–1.95, –0.24]	0.012	–0.47 [–1.37, 0.43]	0.308
PAA	Reference	0.960	–0.45 [–1.51, 0.62]	0.411	–0.55 [–1.60, 0.51]	0.310	0.20 [–0.91, 1.31]	0.723
GAA	Reference	<0.001	0.46 [–0.18, 1.10]	0.163	0.29 [–0.34, 0.93]	0.366	1.80 [1.13, 2.47]	<0.001
Wine Years								
IEAA	Reference	0.589	–0.40 [–1.19, 0.38]	0.311	–0.47 [–1.26, 0.33]	0.248	–0.11 [–0.94, 0.73]	0.805
EEAA	Reference	0.058	–1.01 [–1.87, –0.16]	0.020	–0.31 [–1.18, 0.55]	0.480	–1.03 [–1.94, –0.12]	0.026
PAA	Reference	0.164	–1.09 [–2.14, –0.04]	0.042	–1.26 [–2.32, –0.19]	0.021	–0.50 [–1.62, 0.62]	0.381
GAA	Reference	0.026	–0.01 [–0.66, 0.63]	0.966	0.74 [0.09, 1.39]	0.026	0.59 [–0.10, 1.27]	0.092
Total Alcohol Years								
IEAA	Reference	0.011	–0.51 [–1.40, 0.37]	0.257	–0.50 [–1.41, 0.40]	0.276	–1.28 [–2.22, –0.33]	0.008
EEAA	Reference	0.028	–1.25 [–2.22, –0.29]	0.011	–1.50 [–2.49, –0.51]	0.003	–1.31 [–2.34, –0.28]	0.013
PAA	Reference	0.979	–0.59 [–1.79, 0.60]	0.329	–0.62 [–1.84, 0.61]	0.323	–0.14 [–1.41, 1.14]	0.833
GAA	Reference	<0.001	0.51 [–0.20, 1.22]	0.161	0.71 [–0.02, 1.44]	0.058	2.33 [1.57, 3.09]	<0.001

Results are adjusted for chronological age, sex, race, center, education, pack years of smoking, BMI, and physical activity. Beta coefficients represent the gain in EAA for each alcohol category compared to non-drinkers. Abbreviations: IEAA: intrinsic epigenetic age acceleration; EEAA: extrinsic epigenetic age acceleration; PAA: PhenoAge acceleration; GAA: GrimAge acceleration.

Supplementary Table 4. Analysis results for the association between categorized alcohol consumption and EAA for GEE.

	Non-drinkers		Low		Intermediate		High	
	β [95% CI]	P_{trend}	β [95% CI]	*P*	β [95% CI]	*P*	β [95% CI]	*P*
Beer Years								
IEAA	Reference	0.107	–0.41 [–1.02, 0.19]	0.181	–0.05 [–0.69, 0.59]	0.873	–0.72 [–1.43, 0.02]	0.043
EEAA	Reference	0.139	–0.69 [–1.41, 0.04]	0.064	–0.61 [–1.35, 0.12]	0.103	–0.57 [–1.36, 0.22]	0.155
PAA	Reference	0.421	–0.84 [–1.66, –0.02]	0.046	–0.09 [–0.96, 0.79]	0.847	0.31 [–0.60, 1.22]	0.508
GAA	Reference	<0.001	0.45 [–0.04, 0.95]	0.071	0.59 [0.05, 1.14]	0.033	1.82 [1.24, 2.40]	<0.001
Liquor Years								
IEAA	Reference	0.261	–0.08 [–0.69, 0.52]	0.788	–0.47 [–1.08, 0.15]	0.136	–0.23 [–0.88, 0.41]	0.478
EEAA	Reference	0.273	–0.39 [–1.10, 0.31]	0.271	–0.62 [–1.30, 0.06]	0.074	–0.24 [–0.98, 0.51]	0.530
PAA	Reference	0.203	–0.16 [–0.98, 0.65]	0.700	0.08 [–0.76, 0.92]	0.851	0.64 [–0.23, 1.50]	0.149
GAA	Reference	<0.001	0.44 [–0.03, 0.90]	0.067	0.44 [–0.07, 0.95]	0.091	1.48 [0.90, 2.06]	<0.001
Wine Years								
IEAA	Reference	0.639	–0.55 [–1.12, 0.02]	0.061	–0.12 [–0.76, 0.52]	0.713	–0.19 [–0.82, 0.45]	0.564
EEAA	Reference	0.022	–1.08 [–1.79, –0.36]	0.003	–0.47 [–1.19, 0.25]	0.201	–0.99 [–1.76, –0.22]	0.012
PAA	Reference	0.242	–0.87 [–1.69, –0.06]	0.035	–0.56 [–1.40, 0.27]	0.185	–0.48 [–1.39, 0.42]	0.296
GAA	Reference	0.051	–0.01 [–0.51, 0.50]	0.982	0.43 [–0.06, 0.92]	0.088	0.50 [–0.09, 1.08]	0.096
Total Alcohol Years								
IEAA	Reference	0.145	–0.37 [–1.04, 0.29]	0.272	–0.15 [–0.86, 0.55]	0.667	–0.66 [–1.41, 0.08]	0.081
EEAA	Reference	0.061	–0.67 [–1.48, 0.13]	0.100	–0.78 [–1.59, 0.04]	0.061	–0.89 [–1.75, –0.03]	0.042
PAA	Reference	0.296	–0.14 [–1.05, 0.77]	0.759	0.07 [–0.86, 1.00]	0.882	0.42 [–0.56, 1.40]	0.401
GAA	Reference	<0.001	0.50 [–0.05, 1.05]	0.072	0.66 [0.08, 1.24]	0.025	1.93 [1.31, 2.56]	<0.001

Results are adjusted for chronological age, sex, race, center, education, pack years of smoking, BMI, and physical activity. Beta coefficients represent the gain in EAA for each alcohol category compared to non-drinkers. Abbreviations: IEAA: intrinsic epigenetic age acceleration; EEAA: extrinsic epigenetic age acceleration; PAA: PhenoAge acceleration; GAA: GrimAge acceleration.

Supplementary Table 5. Analysis results for the association between the cumulative amount of absolute alcohol consumed and EAA at examination years 15 and 20.

	Year 15		Year 20		GEE	
	β [95% CI]	*P*	β [95% CI]	*P*	β [95% CI]	*P*
IEAA	0.01 [–0.03, 0.04]	0.752	–0.02 [–0.05, 0.01]	0.166	–0.01 [–0.04, 0.02]	0.447
EEAA	–0.01 [–0.05, 0.04]	0.761	–0.01 [–0.05, 0.02]	0.409	–0.01 [–0.04, 0.02]	0.412
PAA	0.04 [–0.01, 0.09]	0.140	0.02 [–0.03, 0.06]	0.462	0.02 [–0.01, 0.06]	0.224
GAA	0.05 [0.01, 0.08]	0.005	0.05 [0.03, 0.08]	<0.001	0.05 [0.01, 0.08]	0.013

Results are adjusted for chronological age, sex, race, center, education, pack years of smoking, BMI, and physical activity. Beta coefficients represent the gain in EAA for each additional 5 absolute alcohol years. Abbreviations: IEAA: intrinsic epigenetic age acceleration; EEAA: extrinsic epigenetic age acceleration; PAA: PhenoAge acceleration; GAA: GrimAge acceleration.

Supplementary Table 6. Analysis results for the association between weekly alcohol consumption and EAA at examination years 15 and 20.

	Year 15		Year 20		GEE	
	β [95% CI]	*P*	β [95% CI]	*P*	β [95% CI]	*P*
Beer						
IEAA	0.00 [–0.04, 0.04]	0.992	0.01 [–0.03, 0.05]	0.552	0.01 [–0.03, 0.05]	0.723
EEAA	0.01 [–0.03, 0.06]	0.564	0.01 [–0.03, 0.05]	0.721	0.01 [–0.02, 0.04]	0.490
PAA	0.01 [–0.04, 0.07]	0.616	0.00 [–0.05, 0.05]	0.982	0.01 [–0.03, 0.04]	0.747
GAA	0.01 [–0.03, 0.04]	0.667	0.00 [–0.03, 0.04]	0.774	0.01 [–0.06, 0.07]	0.866
Liquor						
IEAA	0.05 [–0.04, 0.13]	0.266	0.02 [–0.04, 0.08]	0.549	0.03 [–0.02, 0.07]	0.270
EEAA	0.00 [–0.10, 0.09]	0.956	0.03 [–0.04, 0.10]	0.392	0.02 [–0.04, 0.07]	0.551
PAA	0.01 [–0.11, 0.12]	0.924	0.05 [–0.04, 0.13]	0.269	0.03 [–0.02, 0.09]	0.243
GAA	0.07 [0.01, 0.14]	0.034	0.06 [0.01, 0.11]	0.015	0.06 [0.00, 0.13]	0.048
Wine						
IEAA	0.02 [–0.07, 0.12]	0.655	0.03 [–0.04, 0.09]	0.458	0.02 [–0.02, 0.07]	0.307
EEAA	0.03 [–0.08, 0.14]	0.610	0.01 [–0.06, 0.08]	0.750	0.01 [–0.05, 0.08]	0.680
PAA	0.02 [–0.11, 0.15]	0.787	0.02 [–0.07, 0.11]	0.624	0.02 [–0.06, 0.10]	0.634
GAA	0.00 [–0.08, 0.07]	0.940	0.03 [–0.02, 0.09]	0.223	0.02 [–0.04, 0.08]	0.517
Total Alcohol						
IEAA	0.01 [–0.02, 0.04]	0.528	0.02 [–0.01, 0.04]	0.284	0.01 [–0.01, 0.04]	0.290
EEAA	0.01 [–0.03, 0.05]	0.520	0.01 [–0.02, 0.04]	0.415	0.01 [–0.01, 0.04]	0.329
PAA	0.01 [–0.03, 0.06]	0.543	0.01 [–0.02, 0.05]	0.468	0.01 [–0.02, 0.04]	0.366
GAA	0.02 [–0.01, 0.04]	0.206	0.02 [0.00, 0.05]	0.058	0.02 [–0.02, 0.06]	0.371

Results are adjusted for chronological age, sex, race, center, education, pack years of smoking, BMI, and physical activity. Beta coefficients represent the gain in EAA for each additional weekly drink. Abbreviations: IEAA: intrinsic epigenetic age acceleration; EEAA: extrinsic epigenetic age acceleration; PAA: PhenoAge acceleration; GAA: GrimAge acceleration.

Supplementary Table 7. Analysis results for the association between cumulative alcohol consumption and telomere length derived from DNA methylation at examination years 15 and 20.

	Year 15		Year 20		GEE	
	β [95% CI]	*P*	β [95% CI]	*P*	β [95% CI]	*P*
Beer Years	–0.00 [–0.01, 0.00]	0.353	–0.00 [–0.00, 0.00]	0.807	–0.00 [–0.01, 0.00]	0.508
Liquor Years	–0.00 [–0.01, 0.01]	0.643	–0.01 [–0.02, 0.00]	0.081	–0.01 [–0.01, 0.00]	0.196
Wine Years	–0.01 [–0.02, 0.01]	0.385	–0.01 [–0.01, 0.00]	0.182	–0.01 [–0.02, 0.00]	0.214
Total Alcohol Years	–0.00 [–0.01, 0.00]	0.270	–0.00 [–0.00, 0.00]	0.209	–0.00 [–0.01, 0.00]	0.167
Recent Binge	–0.01 [–0.04, 0.01]	0.325	–0.03 [–0.06, –0.01]	0.015	–0.02 [–0.04, 0.00]	0.021
Recent Binge Quantity	–0.00 [–0.00, 0.00]	0.531	–0.00 [–0.01, 0.00]	0.019	–0.00 [–0.01, 0.00]	0.069

Results are adjusted for chronological age, sex, race, center, education, pack years of smoking, BMI, and physical activity. Beta coefficients represent the gain in telomere length for each additional 5 alcohol years, for participants who binge drank in the past 30 days, or for each additional day of binge drinking in the past 30 days.

Supplementary Table 8. Analysis results for the association between recent binge drinking and EAA at examination years 15 and 20 among participants with complete follow-up.

	Year 15		Year 20		GEE	
	β [95% CI]	*P*	β [95% CI]	*P*	β [95% CI]	*P*
Recent Binge						
IEAA	–0.21 [–0.91, 0.48]	0.551	–0.39 [–1.13, 0.34]	0.293	–0.30 [–0.83, 0.23]	0.267
EEAA	–0.33 [–1.15, 0.50]	0.438	–0.81 [–1.63, 0.00]	0.051	–0.56 [–1.19, 0.06]	0.078
PAA	0.48 [–0.47, 1.43]	0.325	–0.03 [–1.03, 0.97]	0.952	0.22 [–0.52, 0.97]	0.555
GAA	1.17 [0.60, 1.74]	<0.001	1.28 [0.69, 1.87]	<0.001	1.21 [0.75, 1.68]	<0.001
Recent Binge Quantity						
IEAA	–0.02 [–0.11, 0.07]	0.718	–0.02 [–0.13, 0.10]	0.792	–0.02 [–0.13, 0.09]	0.763
EEAA	–0.05 [–0.16, 0.05]	0.329	0.01 [–0.12, 0.14]	0.913	–0.03 [–0.13, 0.07]	0.555
PAA	–0.01 [–0.13, 0.11]	0.889	0.11 [–0.05, 0.27]	0.173	0.03 [–0.08, 0.15]	0.577
GAA	0.09 [0.01, 0.16]	0.024	0.24 [0.14, 0.33]	<0.001	0.15 [0.06, 0.23]	0.001

Results are adjusted for chronological age, sex, race, center, education, pack years of smoking, BMI, and physical activity. Beta coefficients for recent binge represents the gain in EAA for participants who binge drank in the past 30 days and the beta coefficients for recent binge quantity represents the gain in EAA for each additional day of binge drinking in the past 30 days. Abbreviations: IEAA: intrinsic epigenetic age acceleration; EEAA: extrinsic epigenetic age acceleration; PAA: PhenoAge acceleration; GAA: GrimAge acceleration. Number of participants with complete follow up: $n_{Y15} = 890$; $n_{Y20} = 820$.

Supplementary Table 9. Analysis results for the association between cumulative alcohol consumption and GAA at examination years 15 and 20, mutually adjusted for recent binge drinking.

	Year 15		Year 20		GEE	
	β [95% CI]	*P*	β [95% CI]	*P*	β [95% CI]	*P*
Beer Years						
Cumulative Consumption	0.02 [–0.10, 0.13]	0.787	0.08 [–0.01, 0.17]	0.081	0.05 [–0.09, 0.19]	0.467
Recent Binge	1.19 [0.60, 1.78]	<0.001	1.38 [0.79, 1.97]	<0.001	1.27 [0.74, 1.80]	<0.001
Liquor Years						
Cumulative Consumption	0.20 [–0.02, 0.42]	0.082	0.22 [0.04, 0.39]	0.016	0.20 [0.00, 0.39]	0.046
Recent Binge	1.08 [0.52, 1.63]	<0.001	1.35 [0.77, 1.93]	<0.001	1.21 [0.74, 1.68]	<0.001
Wine Years						
Cumulative Consumption	–0.04 [–0.30, 0.22]	0.762	–0.02 [–0.20, 0.16]	0.828	–0.02 [–0.22, 0.17]	0.807
Recent Binge	1.24 [0.70, 1.79]	<0.001	1.57 [0.99, 2.15]	<0.001	1.39 [0.93, 1.85]	<0.001
Total Alcohol Years						
Cumulative Consumption	0.03 [–0.05, 0.12]	0.433	0.07 [0.00, 0.14]	0.037	0.05 [–0.04, 0.15]	0.271
Recent Binge	1.11 [0.51, 1.71]	<0.001	1.28 [0.67, 1.89]	<0.001	1.19 [0.65, 1.74]	<0.001

Results are adjusted for chronological age, sex, race, center, education, pack years of smoking, BMI, and physical activity. Beta coefficients for the cumulative alcohol variables represent the gain in GAA for each additional 5 alcohol years and beta coefficients for recent binge represents the gain in GAA for participants who binge drank in the past 30 days.

Supplementary Table 10. Summary statistics for cumulative alcohol consumption and recent binge drinking by chronological age quartile.

	Year 15	Year 20
Beer Years		
Quartile 1	5.7 (10.9)	8.1 (15.4)
Quartile 2	6.9 (12.3)	8.6 (12.9)
Quartile 3	6.2 (11.9)	6.5 (13.7)
Quartile 4	5.1 (10.4)	7.1 (17.8)
Liquor Years		
Quartile 1	2.3 (6.2)	3.5 (9.4)
Quartile 2	1.9 (4.9)	2.7 (5.7)
Quartile 3	1.9 (4.4)	2.6 (6.1)
Quartile 4	2.3 (5.8)	3.1 (7.2)
Wine Years		
Quartile 1	1.2 (2.5)	1.9 (3.6)
Quartile 2	2.2 (4.5)	3.4 (7.3)
Quartile 3	2.5 (4.9)	3.5 (7.2)
Quartile 4	2.9 (5.5)	4.8 (8.7)
Total Alcohol Years		
Quartile 1	9.2 (16.0)	13.5 (22.8)
Quartile 2	11.0 (15.8)	14.6 (17.2)
Quartile 3	10.5 (15.3)	12.7 (19.5)
Quartile 4	10.4 (15.5)	15.1 (25.8)
Recent Binge		
Quartile 1	64 (26.5)	55 (24.7)
Quartile 2	73 (29.7)	66 (29.5)
Quartile 3	81 (27.6)	64 (23.6)
Quartile 4	41 (16.9)	39 (17.2)

All statistics are shown as mean and standard deviation, expect for recent binge drinking, which are shown as number of participants and percentages.

Supplementary Table 11. Interaction and stratified analysis results for the association between cumulative alcohol consumption and GAA at examination years 15 and 20 by strata of chronological age quartiles among participants with complete follow-up.

	Year 15		Year 20		GEE	
	$B_{alcohol}$ [95% CI]	*P*	$B_{alcohol}$ [95% CI]	*P*	$B_{alcohol}$ [95% CI]	*P*
Beer Years	**–0.04 [–0.07, –0.02]**	**0.002***	**–0.04 [–0.06, –0.02]**	**<0.001***	**–0.03 [–0.05, –0.01]**	**<0.001***
Quartile 1	0.34 [0.12, 0.56]	0.003	0.29 [0.12, 0.47]	0.001	0.30 [0.15, 0.45]	<0.001
Quartile 2	0.33 [0.12, 0.55]	0.002	0.37 [0.14, 0.59]	0.001	0.34 [0.16, 0.51]	<0.001
Quartile 3	–0.09 [–0.31, 0.13]	0.408	0.28 [0.11, 0.44]	0.001	0.11 [–0.05, 0.27]	0.178
Quartile 4	–0.04 [–0.29, 0.21]	0.744	–0.17 [–0.34, 0.00]	0.045	–0.16 [–0.35, 0.03]	0.108
Liquor Years	**–0.03 [–0.08, 0.03]**	**0.360**	**0.00 [–0.04, 0.05]**	**0.872**	**–0.01 [–0.05, 0.03]**	**0.670**
Quartile 1	0.41 [0.02, 0.80]	0.039	0.17 [–0.13, 0.48]	0.261	0.25 [–0.06, 0.57]	0.113
Quartile 2	0.42 [–0.20, 1.05]	0.185	0.13 [–0.43, 0.69]	0.642	0.24 [–0.36, 0.84]	0.430
Quartile 3	0.03 [–0.50, 0.56]	0.907	0.44 [0.07, 0.80]	0.018	0.25 [–0.19, 0.69]	0.259
Quartile 4	0.25 [–0.16, 0.66]	0.231	0.27 [–0.11, 0.65]	0.159	0.26 [–0.11, 0.63]	0.173
Wine Years	**–0.05 [–0.13, 0.03]**	**0.202**	**–0.07 [–0.13, –0.01]**	**0.022***	**–0.04 [–0.10, 0.01]**	**0.144**
Quartile 1	0.48 [–0.45, 1.42]	0.308	0.61 [–0.12, 1.35]	0.102	0.54 [–0.10, 1.19]	0.100
Quartile 2	0.04 [–0.49, 0.58]	0.870	0.06 [–0.28, 0.40]	0.717	0.04 [–0.23, 0.30]	0.794
Quartile 3	–0.06 [–0.62, 0.50]	0.831	0.19 [–0.16, 0.54]	0.281	0.08 [–0.19, 0.34]	0.570
Quartile 4	0.00 [–0.48, 0.47]	0.990	–0.22 [–0.57, 0.12]	0.208	–0.15 [–0.56, 0.25]	0.457
Total Alcohol Years	**–0.03 [–0.05, –0.01]**	**0.004***	**–0.03 [–0.04, –0.02]**	**<0.001***	**–0.02 [–0.04, 0.01]**	**0.006***
Quartile 1	0.25 [0.09, 0.40]	0.002	0.20 [0.07, 0.32]	0.002	0.21 [0.09, 0.33]	0.001
Quartile 2	0.22 [0.06, 0.38]	0.007	0.22 [0.06, 0.38]	0.009	0.21 [0.06, 0.35]	0.006
Quartile 3	–0.06 [–0.23, 0.11]	0.502	0.21 [0.10, 0.33]	<0.001	0.10 [–0.01, 0.20]	0.081
Quartile 4	0.02 [–0.15, 0.19]	0.789	–0.08 [–0.20, 0.03]	0.168	–0.06 [–0.22, 0.09]	0.416

*Interaction terms with $P \leq 0.05$. Bolded values represent the beta coefficient [95% CI] and *P* for the joint association between alcohol years and chronological age. Results are adjusted for sex, race, center, education, pack years of smoking, BMI, and physical activity. Beta coefficients represent the gain in EAA for each additional 5 alcohol years. Number of participants with complete follow up: n_{Y15} = 890; n_{Y20} = 820.

Supplementary Table 12. Stratified analysis results for the association between cumulative alcohol consumption and GAA at examination years 15 and 20 by strata of chronological age quartiles, mutually adjusted for recent binge drinking.

	Year 15		Year 20		GEE	
	$B_{alcohol}$ [95% CI]	*P*	$B_{alcohol}$ [95% CI]	*P*	$B_{alcohol}$ [95% CI]	*P*
Beer Years	**–0.04 [–0.07, –0.02]**	**0.002***	**–0.04 [–0.05, –0.02]**	**<0.001***	**–0.03 [–0.04, –0.01]**	**<0.001***
Quartile 1	0.22 [–0.04, 0.47]	0.092	0.19 [0.01, 0.37]	0.044	0.20 [0.03, 0.36]	0.020
Quartile 2	0.23 [0.01, 0.45]	0.038	0.32 [0.08, 0.56]	0.008	0.26 [0.08, 0.44]	0.006
Quartile 3	–0.17 [–0.39, 0.05]	0.129	0.24 [0.07, 0.40]	0.006	0.05 [–0.09, 0.19]	0.469
Quartile 4	–0.14 [–0.40, 0.13]	0.311	–0.20 [–0.37, –0.03]	0.023	–0.20 [–0.36, –0.04]	0.017
Liquor Years	**–0.03 [–0.08, 0.03]**	**0.337**	**–0.01 [–0.05, 0.03]**	**0.619**	**–0.01 [–0.05, 0.03]**	**0.516**
Quartile 1	0.32 [–0.07, 0.71]	0.107	0.09 [–0.19, 0.37]	0.543	0.16 [–0.12, 0.45]	0.263
Quartile 2	0.00 [–0.49, 0.49]	0.999	0.10 [–0.36, 0.57]	0.669	0.03 [–0.36, 0.42]	0.834
Quartile 3	–0.13 [–0.65, 0.40]	0.638	0.27 [–0.11, 0.64]	0.161	0.08 [–0.37, 0.53]	0.725
Quartile 4	0.35 [–0.08, 0.77]	0.108	0.21 [–0.18, 0.60]	0.288	0.26 [–0.12, 0.64]	0.179
Wine Years	**–0.03 [–0.11, 0.04]**	**0.377**	**–0.06 [–0.12, –0.01]**	**0.030***	**–0.03 [–0.08, 0.02]**	**0.207**
Quartile 1	0.20 [–0.70, 1.10]	0.658	0.28 [–0.44, 1.00]	0.441	0.24 [–0.40, 0.87]	0.465
Quartile 2	–0.19 [–0.70, 0.31]	0.453	–0.02 [–0.36, 0.32]	0.908	–0.08 [–0.34, 0.17]	0.518
Quartile 3	–0.15 [–0.64, 0.35]	0.562	0.08 [–0.23, 0.39]	0.594	–0.02 [–0.24, 0.20]	0.882
Quartile 4	0.06 [–0.39, 0.51]	0.803	–0.24 [–0.57, 0.08]	0.144	–0.14 [–0.51, 0.22]	0.436
Total Alcohol Years	**–0.03 [–0.04, –0.01]**	**0.005***	**–0.03 [–0.04, –0.01]**	**<0.001***	**–0.02 [–0.03, –0.01]**	**0.006***
Quartile 1	0.17 [0.00, 0.35]	0.049	0.13 [–0.01, 0.25]	0.055	0.13 [0.01, 0.26]	0.040
Quartile 2	0.12 [–0.05, 0.29]	0.177	0.22 [0.03, 0.40]	0.025	0.14 [0.00, 0.28]	0.057
Quartile 3	–0.15 [–0.33, 0.03]	0.106	0.17 [0.04, 0.31]	0.012	0.04 [–0.06, 0.13]	0.448
Quartile 4	0.01 [–0.17, 0.19]	0.926	–0.12 [–0.24, 0.01]	0.063	–0.09 [–0.23, 0.06]	0.238

*Interaction terms with $P \leq 0.05$. Bolded values represent the beta coefficient [95% CI] and *P* for the joint association between alcohol years and chronological age. Results are adjusted for sex, race, center, education, pack years of smoking, BMI, physical activity, and recent binge drinking. Beta coefficients represent the gain in GAA for each additional 5 alcohol years.

Supplementary Table 13. Interaction and stratified analysis results for the association between cumulative alcohol consumption and GAA at examination years 15 and 20 by sex.

	Year 15		Year 20		GEE	
	$B_{alcohol}$ [95% CI]	*P*	$B_{alcohol}$ [95% CI]	*P*	$B_{alcohol}$ [95% CI]	*P*
Beer Years	**–0.57 [–0.91, –0.23]**	**0.001***	**–0.52 [–0.80, –0.25]**	**<0.001***	**–0.53 [–0.83, –0.22]**	**<0.001***
Female	0.52 [0.21, 0.82]	0.001	0.56 [0.30 0.82]	<0.001	0.52 [0.23, 0.82]	<0.001
Male	0.09 [–0.03, 0.22]	0.138	0.12 [0.02, 0.22]	0.014	0.11 [–0.03, 0.24]	0.123
Liquor Years	**–0.20 [–0.65, 0.25]**	**0.381**	**–0.08 [–0.46, 0.31]**	**0.691**	**–0.12 [–0.63, 0.39]**	**0.649**
Female	0.34 [–0.01, 0.69]	0.056	0.31 [–0.02, 0.63]	0.062	0.30 [–0.15, 0.75]	0.191
Male	0.32 [0.04, 0.60]	0.025	0.35 [0.14, 0.55]	<0.001	0.33 [0.11, 0.54]	0.004
Wine Years	**0.29 [–0.21, 0.78]**	**0.261**	**–0.23 [–0.59, 0.12]**	**0.202**	**–0.04 [–0.40, 0.32]**	**0.817**
Female	–0.01 [–0.40, 0.37]	0.949	0.29 [0.01, 0.58]	0.046	0.18 [–0.09, 0.46]	0.197
Male	0.22 [–0.14, 0.57]	0.228	0.03 [–0.20, 0.25]	0.824	0.08 [–0.18, 0.33]	0.551
Total Alcohol Years	**–0.18 [–0.37, 0.01]**	**0.060**	**–0.22 [–0.37, –0.06]**	**0.005***	**–0.19 [–0.40, 0.01]**	**0.062**
Female	0.22 [0.06, 0.38]	0.009	0.28 [0.14, 0.42]	<0.001	0.24 [0.05, 0.43]	0.013
Male	0.11 [0.01, 0.20]	0.027	0.11 [0.04, 0.18]	0.003	0.10 [0.01, 0.20]	0.032

*Interaction terms with $P \leq 0.05$. Bolded values represent the beta coefficient [95% CI] and *P* for the joint association between alcohol years and sex. Results are adjusted for chronological age, race, center, education, pack years of smoking, BMI, and physical activity. Beta coefficients represent the gain in GAA for each additional 5 alcohol years.

Supplementary Table 14. Interaction and stratified analysis results for the association between cumulative alcohol consumption and GAA at examination years 15 and 20 by race.

	Year 15		Year 20		GEE	
	**$B_{alcohol}$ [95% CI]**	***P***	**$B_{alcohol}$ [95% CI]**	***P***	**$B_{alcohol}$ [95% CI]**	***P***
Beer Years	**–0.05 [–0.25, 0.15]**	**0.625**	**–0.02 [–0.18, 0.14]**	**0.808**	**–0.02 [–0.30, 0.25]**	**0.861**
Black	0.05 [–0.14, 0.24]	0.592	0.15 [0.01, 0.29]	0.038	0.10 [–0.18, 0.38]	0.487
White	0.12 [–0.01, 0.25]	0.072	0.15 [0.04, 0.27]	0.011	0.13 [0.04, 0.23]	0.007
Liquor Years	**–0.53 [–0.97, –0.09]**	**0.017***	**–0.19 [–0.52, 0.13]**	**0.244**	**–0.27 [–0.65, 0.10]**	**0.153**
Black	0.62 [0.22, 1.02]	0.003	0.41 [0.14, 0.68]	0.003	0.44 [0.11, 0.78]	0.010
White	0.16 [–0.09, 0.41]	0.204	0.23 [0.01, 0.45]	0.044	0.19 [–0.04, 0.42]	0.104
Wine Years	**–0.29 [–1.03, 0.45]**	**0.441**	**0.05 [–0.41, 0.50]**	**0.838**	**–0.01 [–0.81, 0.78]**	**0.973**
Black	0.05 [–0.72, 0.83]	0.893	0.00 [–0.47, 0.46]	0.988	–0.01 [–0.79, 0.77]	0.976
White	0.03 [–0.24, 0.30]	0.838	0.12 [–0.06, 0.31]	0.184	0.09 [–0.10, 0.28]	0.350
Total Alcohol Years	**–0.10 [–0.25, 0.04]**	**0.167**	**–0.03 [–0.14, 0.08]**	**0.570**	**–0.05 [–0.25, 0.15]**	**0.635**
Black	0.12 [–0.03, 0.27]	0.118	0.14 [0.04, 0.25]	0.007	0.12 [–0.08, 0.32]	0.230
White	0.08 [–0.01, 0.17]	0.077	0.12 [0.04, 0.19]	0.003	0.10 [0.03, 0.17]	0.008

*Interaction terms with $P \leq 0.05$. Bolded values represent the beta coefficient [95% CI] and *P* for the joint association between alcohol years and race. Results are adjusted for chronological age, sex, center, education, pack years of smoking, BMI, and physical activity. Beta coefficients represent the gain in GAA for each additional 5 alcohol years.
